# Downregulation of CREB expression in Alzheimer's brain and in Aβ-treated rat hippocampal neurons

**DOI:** 10.1186/1750-1326-6-60

**Published:** 2011-08-19

**Authors:** Subbiah Pugazhenthi, Maorong Wang, Serena Pham, Chun-I Sze, Christopher B Eckman

**Affiliations:** 1 Section of Endocrinology, Veterans Affairs Medical Center, Denver, CO, USA; 2 Department of Medicine, University of Colorado Denver, Aurora, CO, USA; 3 Nanjing Bayi Hospital, Nanjing University of Traditional Chinese Medicine, Nanjing, China; 4 Department of Cell Biology and Anatomy, National Cheng Kung University Medical College, Tainan, Taiwan; 5 MidAtlantic Neonatal Research Institute, Atlantic Health Systems, Morristown, NJ, USA

**Keywords:** Alzheimer's disease, CREB, Oxidative stress, Apoptosis, Tg2576 mice, Laser capture microdissection

## Abstract

**Background:**

Oxidative stress plays an important role in neuronal dysfunction and neuron loss in Alzheimer's brain. Previous studies have reported downregulation of CREB-mediated transcription by oxidative stress and Aβ. The promoter for CREB itself contains cyclic AMP response elements. Therefore, we examined the expression of CREB in the hippocampal neurons of Tg2576 mice, AD post-mortem brain and in cultured rat hippocampal neurons exposed to Aβ aggregates.

**Results:**

Laser Capture Microdissection of hippocampal neurons from Tg2576 mouse brain revealed decreases in the mRNA levels of CREB and its target, BDNF. Immunohistochemical analysis of Tg2576 mouse brain showed decreases in CREB levels in hippocampus and cortex. Markers of oxidative stress were detected in transgenic mouse brain and decreased CREB staining was observed in regions showing abundance of astrocytes. There was also an inverse correlation between SDS-extracted Aβ and CREB protein levels in Alzheimer's post-mortem hippocampal samples. The levels of CREB-regulated BDNF and BIRC3, a caspase inhibitor, decreased and the active cleaved form of caspase-9, a marker for the intrinsic pathway of apoptosis, was elevated in these samples. Exposure of rat primary hippocampal neurons to Aβ fibrils decreased CREB promoter activity. Decrease in CREB mRNA levels in Aβ-treated neurons was reversed by the antioxidant, N-acetyl cysteine. Overexpression of CREB by adenoviral transduction led to significant protection against Aβ-induced neuronal apoptosis.

**Conclusions:**

Our findings suggest that chronic downregulation of CREB-mediated transcription results in decrease of CREB content in the hippocampal neurons of AD brain which may contribute to exacerbation of disease progression.

## Background

Cyclic AMP response element binding protein (CREB) is a constitutively expressed nuclear transcription factor that regulates the expression of genes involved in neuronal survival and function [1-3]. CREB is essential for the formation and retention of memory in several species [4,5]. CREB-mediated gene expression is increased in the hippocampus during LTP [6]. Spatial learning deficits in rats are observed after intra-hippocampal infusion of CREB antisense oligos [7]. CREB is also an important nuclear target that couples neurotrophin-mediated signaling to neuronal survival [8]. CREB undergoes phosphorylation at serine 133 in response to multiple signaling pathways [9,10]. The phosphorylated form of CREB binds to the coactivators, CREB binding protein (CBP) and p300 resulting in the facilitation of target gene expression [11]. We have reported that IGF-I induces CREB-regulated expression of the anti-apoptotic gene, bcl-2 through multiple signaling pathways [12,13].

Previous studies have reported that CREB-mediated gene expression is impaired in Alzheimer's brain. The active form of p90RSK, a critical CREB kinase, is decreased in a transgenic rat AD model [14]. Gong et al [15] reported a decrease in the levels of phosphorylated CREB in hippocampal neurons of PS1/APP double mutant transgenic mice. Treatment of these mice with rolipram, a phosphodiesterase inhibitor that increases CREB phosphorylation results in a significant improvement in cognitive function. In AD post-mortem brain, there is a decrease in the levels of CREB-regulated BDNF [16]. In cultured neurons, Aβ interferes with CREB activation by cAMP and BDNF [17-19]. However, paradoxical CREB activation in conditions associated with AD is also reported [20,21]. We demonstrated that lipid peroxidation products increase the levels of active phosphorylated form of CREB without a parallel increase in CREB-mediated gene expression [22]. We also reported oxidative stress-induced downregulation of CREB-dependent bcl-2 expression in cultured rat hippocampal neurons [23]. In the same study, we showed that hydrogen peroxide decreases CREB protein levels in neurons which were normalized by preincubation with the antioxidant, N acetyl cysteine. The objective of the present study was to determine the expression of CREB in three different models of AD, namely the Tg2576 mouse which expresses human APP with the Swedish mutation, AD post-mortem brain and cultured rat hippocampal neurons exposed to Aβ.

## Results

### Laser capture microdissection (LCM) reveals decrease of CREB mRNA in hippocampal neurons of Tg2576 mouse brain

**Figure 1:**
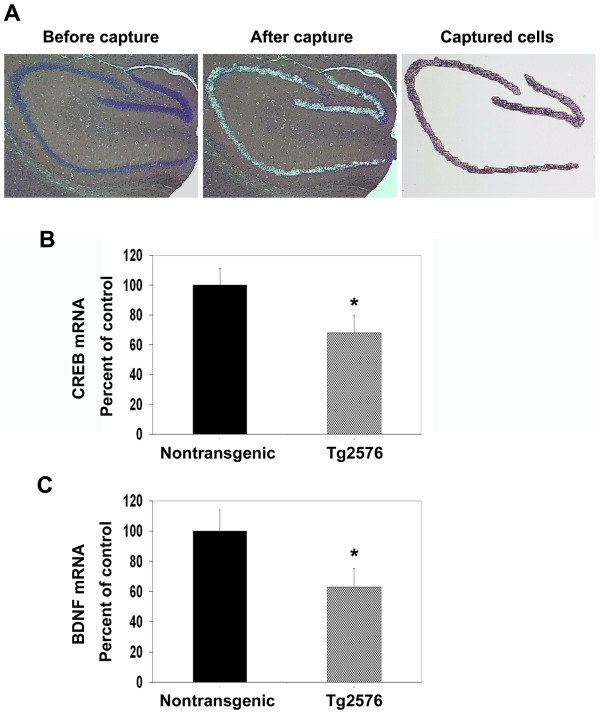
**Laser capture microdissection (LCM) of hippocampal neurons from Tg2576 mouse brain**. Brain sections (10 μM) from Tg2576 transgenic mice along with nontransgenic controls were stained using HistoGene™ Stain, (Arcturus) to identify the neurons. (A) LCM was performed to selectively capture the neurons in CA1, CA3 and dentate gyrus regions onto Capsure LCM macro caps. Captured cells were processed for RNA isolation, RNA amplification and RT-PCR analysis. The mRNA levels of CREB (B) and its target BDNF (C) decreased in Tg2576 hippocampal neurons. *p < 0.01 compared to nontransgenic control.

CREB, a constitutively expressed nuclear transcription factor, is activated by phosphorylation at serine 133. We have previously reported that CREB expression itself is downregulated in cultured rat hippocampal neurons exposed to hydrogen peroxide [23]. The objective of the current study was to determine if oxidative stress generated *in vivo *in Tg2576 mouse brain decreases CREB expression. As hippocampus consists of neuronal as well as glial cells, LCM was performed to determine the expression of CREB specifically in hippocampal neurons (Figure 1A). Following amplification of RNA isolated from captured hippocampal neurons, the mRNA levels of CREB and its target BDNF were determined by real time RT-PCR analysis using Taqman probes. There was a 33% decrease in the mRNA levels of CREB in hippocampal neurons of Alzheimer's mouse brain (Figure 1B). Similarly, 38% decrease in the expression of BDNF was also observed (Figure 1C). The promoter for BDNF contains cyclic AMP response elements and is positively regulated by CREB.

### Decrease in CREB protein levels of Alzheimer's mouse hippocampal neurons

**Figure 2:**
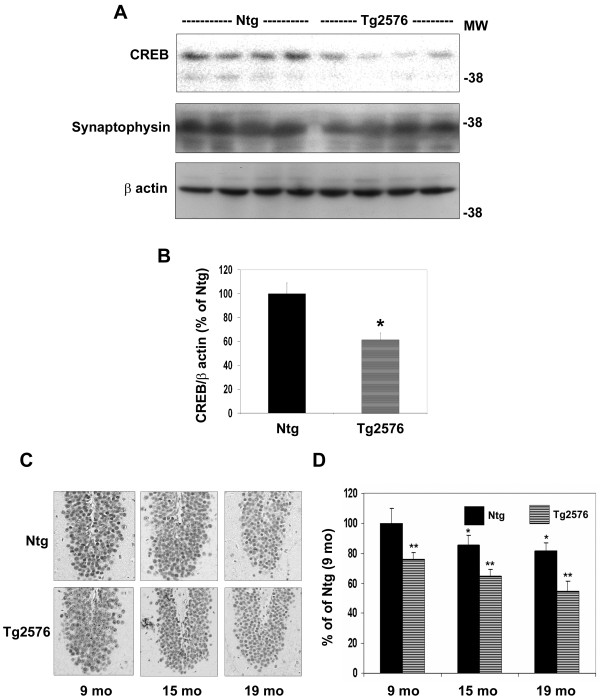
**CREB protein levels in hippocampal neurons of mouse brain**. (A) Western blot analyses of CREB and synaptophysin were carried out with mouse hippocampal samples from 15-19 month old nontransgenic (Ntg) and Tg2576 mice. The blots were reprobed for β Actin. (B) The band intensities were quantitated by scanning. * p < 0.01 vs nontransgenic. (C) Hippocampal sections from Tg2576 mice of different ages were immunostained for CREB. Representative images from dentate gyrus regions are presented. (D) The intensities of CREB stain in hippocampal neurons of different regions were quantitated by ImagepPro analysis. * p < 0.05 vs nontransgenic control of 9 month old mice. ** p < 0.01 compared to nontransgenic control at respective age.

Western blot analysis of CREB and correction of band intensities with β actin levels showed 40% decrease (p < 0.01) in Tg2576 mouse hippocampus as compared to control samples (Figure 2A and 2B). The levels of synaptophysin, a neuronal protein, did not change significantly between the two groups. When CREB intensities were corrected for synaptophysin levels significant decrease (p < 0.01) was observed. Immunohistochemical staining of brain sections for CREB revealed gradual decrease (p < 0.05) in CREB protein with aging from 9 to 19 months in both groups of mice. More pronounced decreases were observed in all the age groups of Tg2576 mice. The images for the dentate gyrus region alone are presented (Figure 2C). Quantitation of CREB signals in hippocampal neurons by ImagePro analysis revealed 23-32% decrease of CREB levels in Tg2576 mouse hippocampal neurons at 9, 15, and 19 months of age when compared to nontransgenic mice at respective ages (Figure 2C). These findings further confirmed that CREB expression is decreased in the hippocampal neurons of Alzheimer's mouse brain and suggested the roles of Aβ deposition and aging.

### Markers of oxidative stress in Tg2576 mouse brain

**Figure 3:**
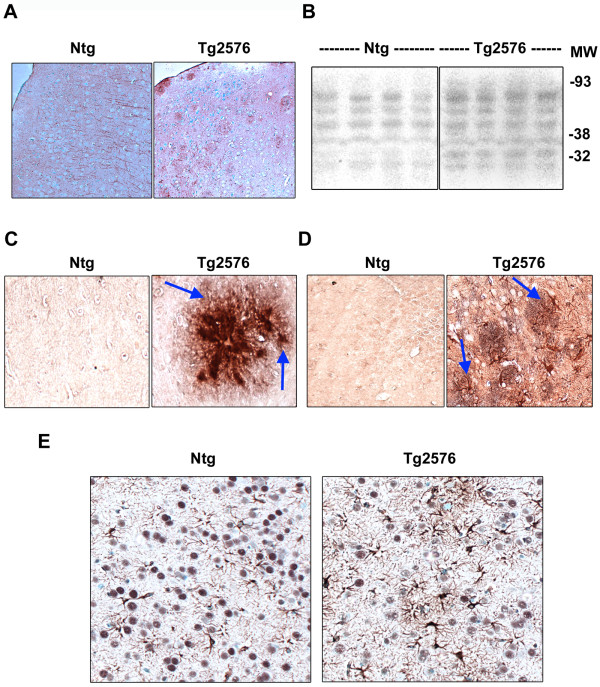
**Markers of oxidative stress in Alzheimer's mouse brain**. (A) Brain sections of 15-17 month old nontransgenic (Ntg) and Tg2576 mice were immunostained for HNE with NovaRED and counterstained with methyl green. (B) Western blot analysis of mouse brain samples for protein bound HNE was performed. (C) Double immunostaining of mouse brain sections was carried out with monoclonal antibodies to Aβ (4G8) and polyclonal antibody for MDA. The chromogens were NovaRED for Aβ and DAB for MDA. (D) Double immunostaining of mouse brain sections was carried out with monoclonal antibody to GFAP (NovaRED) and polyclonal antibody to MDA (DAB). (E) Double immunostaining was performed with monoclonal antibody to GFAP (NovaRED) and polyclonal antibody for CREB (DAB). Representative images are provided. Markers of oxidative stress were elevated in Tg2576 mouse brain.

Oxidative stress could be one of the potential causes of decreased CREB expression observed in Alzheimer's mouse brain. We have previously reported that CREB expression is decreased in cultured rat hippocampal neurons exposed to hydrogen peroxide which is prevented when the neurons are pretreated with the antioxidant, N-acetyl cysteine [23]. Therefore, we examined the mouse brain samples for the presence of markers of oxidative stress. Aβ deposition is known to generate free radicals in AD brain resulting in lipid peroxidation. One such unsaturated aldehyde is 4-hydroxynonenal (HNE). Increased staining for HNE was seen in the regions of Aβ deposition in Tg2576 mouse brain (Figure 3A). Protein bound HNE levels also increased in these samples as shown by increases in the intensities of multiple bands in the Western blot analysis (Figure 3B). Dual immunohistochemical analysis showed colocalization of malondialdehyde (MDA), another marker for oxidative stress, stained with DAB (arrows) around NovaRed-stained Aβ deposits (Figure 3C). Astrocytosis as seen in AD is also likely to contribute to the generation of oxidative stress through cytokine release [24]. Therefore, mouse brain sections were double-immunostained for GFAP, a marker for astrocyte with NovaRed and for MDA with DAB (Figure 3D). Increase in MDA levels were seen around astrocytes as shown with a representative image. These observations suggest a pro-oxidative environment in AD brain that is likely to cause CREB downregulation. Next, double immunostaining for GFAP (astrocyte) and CREB was carried out and a representative image is shown in Figure 3E. Decreases in CREB content were associated with abundance of GFAP in Tg2576 mouse brain. ImagePro analysis of CREB content in multiple fields further showed a mean decrease of 42% (p < 0.001) in high GFAP areas of Tg2576 mouse cortex as compared to low GFAP areas in nontransgenic mouse brain. These observations suggest a potential relationship between oxidative stress and CREB downregulation.

### Inverse correlation between Aβ deposition and CREB content

**Figure 4:**
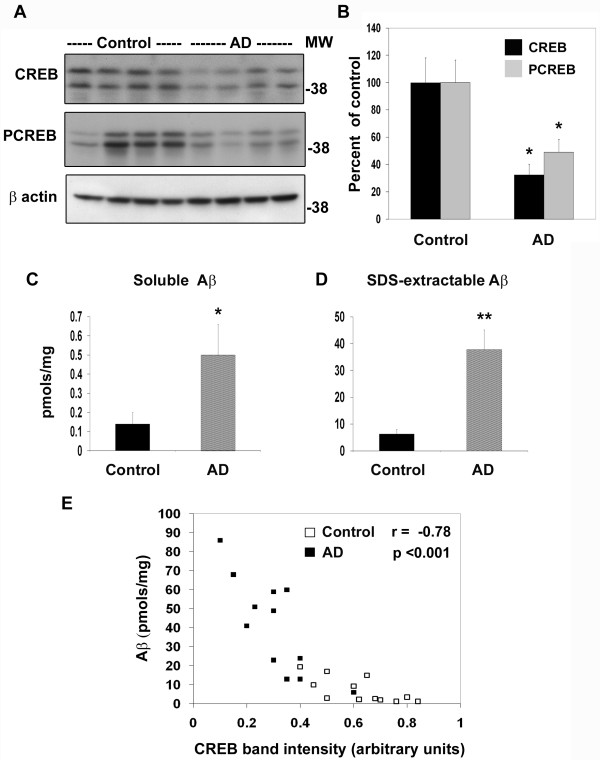
**Decrease in CREB protein in AD-postmortem brain**. (A) Post-mortem hippocampal samples (12 each) of AD cases and age-matched controls with equal protein content were electrophoresed, transferred, and immunoprobed with antibodies to CREB, phospho CREB (PCREB) and β actin. Representative blots are shown. (B) Band intensities of CREB and PCREB were quantitated and corrected for β actin for 24 samples. Total and phospho CREB levels were significantly low in AD brain. *P < 0.01 compared to control. (C, D) Aβ 1-42 levels were determined in soluble and SDS-extractable fractions of post-mortem samples by sandwich ELISA. Elevated levels of soluble and SDS-extractible Aβ were observed in AD brain. *p < 0.01; **p < 0.001 vs control. (E) When the levels of SDS-soluble Aβ were plotted against CREB band intensities, an inverse correlation between the two parameters was observed. Control values are shown as open squares and AD values as filled squares.

Having observed downregulation of CREB expression in Tg2576 mouse brain, next we examined AD post-mortem samples to determine the levels of CREB. Western blot analysis showed decrease of CREB content in AD hippocampal samples when compared to age-matched controls (Figure 4A). Phosphorylated form of CREB also decreased significantly in AD brain which is likely to be the result of decrease in the levels of CREB protein itself. Quantitation of band intensities revealed a 52% decrease in CREB levels in AD hippocampi (Figure 4B). We also determined the levels of Aβ (1-42) in the post-mortem samples using a sandwich ELISA. There was a 257% increase in the levels of soluble Aβ in AD hippocampal samples (Figure 4C). In the case of SDS-extractable Aβ (1-42), the increase was significantly more (505%; Figure 4D). When SDS-soluble Aβ levels were plotted against CREB band intensities, significant (p < 0.001) inverse relationship between the two was observed with a correlation co-efficient of 0.78 (Figure 4E) suggesting that Aβ deposition may lead to decrease in CREB content in AD brain. No significant correlation between soluble Aβ and CREB levels was observed (results not shown).

### Decreased expression of CREB-regulated proteins and markers of oxidative stress in AD brain

**Figure 5:**
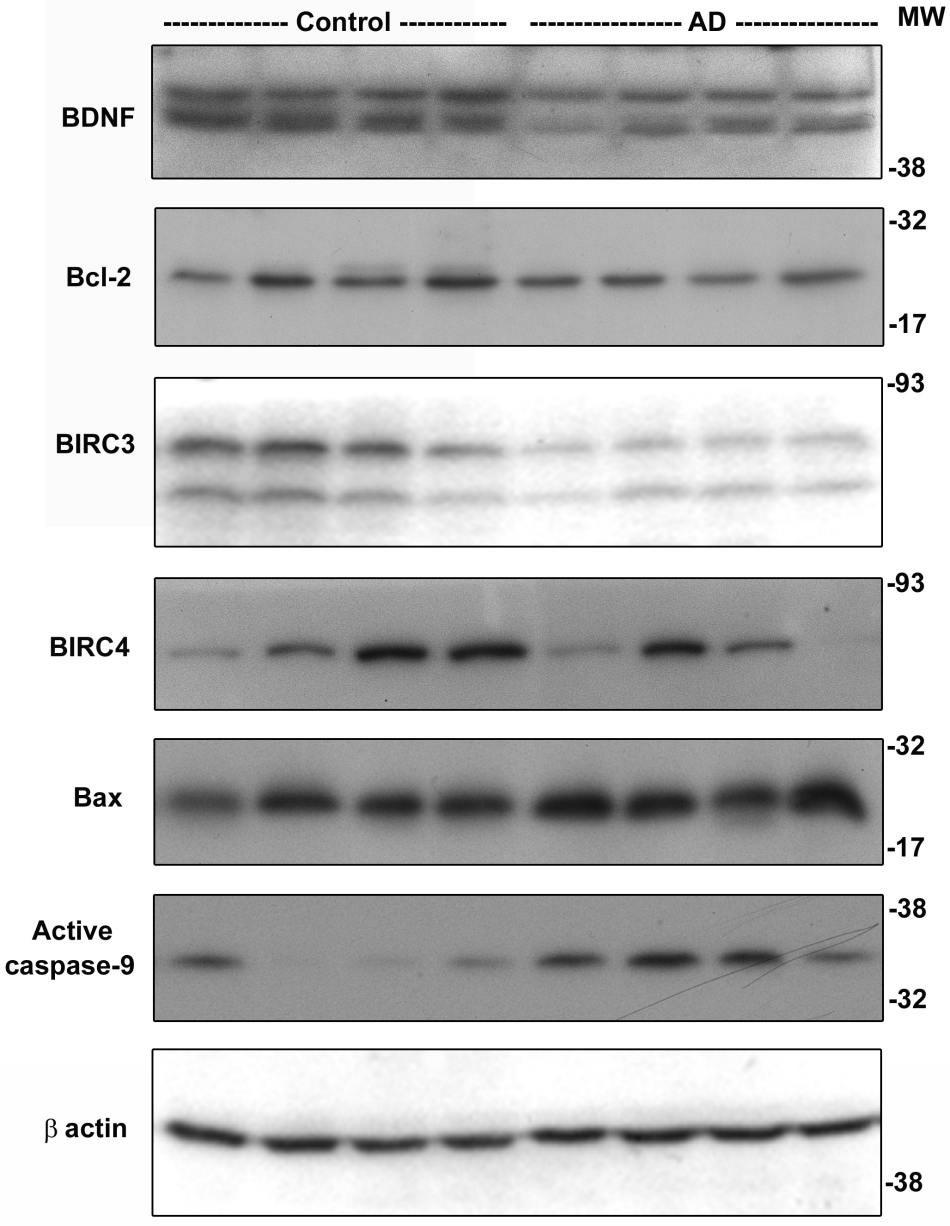
**Markers of CREB function and apoptosis in AD-post mortem brain**. Post-mortem hippocampal samples (8 each) of AD cases and age-matched controls with equal protein content were electrophoresed, transferred, and immunoprobed with antibodies for markers of CREB function and apoptosis. The blots were reprobed for β actin. Representative images are provided. Decreases in CREB-regulated proteins that improve neuronal survival and increases in the levels of the active form of caspase-9, a marker for the intrinsic pathway of apoptosis were observed in AD-post mortem brain.

**Figure 6:**
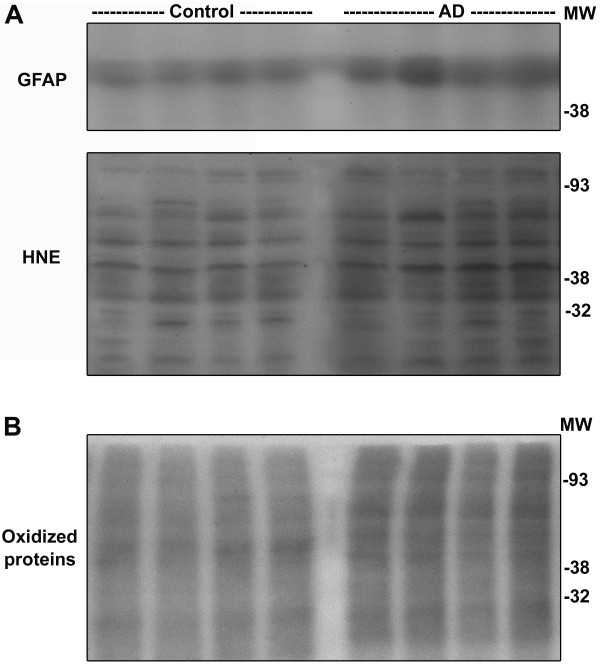
**Markers of oxidative stress in AD-post mortem brain**. (A) Post-mortem hippocampal samples (8 each) of AD cases and age-matched controls with equal protein content were immunoblotted for GFAP and HNE, markers of oxidative stress. Representative images are provided. There was accumulation of markers of oxidative stress in AD brain. (B) Post-mortem samples were treated with 2,4-dinitrophenylhydrazine (DNPH) to form DNP-hydrazone. DNP-derivatized protein samples were electrophoresed and immunoblotted with anti-DNP antibody. Oxidative modification of proteins was elevated in AD brain samples.

Next we examined the expression of a panel of CREB-dependent proteins that are involved in neuronal function and survival. Significant decreases in AD brain were observed for BDNF (31%; p < 0.01), Bcl-2 (23%; p < 0.05), BIRC3 (44% p < 0.01) and BIRC4 (32%; p < 0.01) when compared to age-matched controls (Figure 5). Bcl-2 is an anti-apoptotic protein that maintains the integrity of mitochondrial membrane. BIRC3 (c-IAP2) and BIRC4 (XIAP) are caspase inhibitors that play important roles in neuronal survival. In addition, the levels of proapoptotic Bax increased by 38% (p < 0.05), probably as a result of oxidative stress. Modulation of proteins in apoptosis pathway, together, resulted in 150% increase in the levels of active cleaved form of caspase-9, a marker for the intrinsic pathway of apoptosis. Markers of oxidative stress were also examined with post mortem samples. Increases in the levels GFAP (astrocyte) and protein-bound HNE were observed in AD brain (Figure 6A). Furthermore, Oxyblot analysis showed increases in the intensities of multiple bands (Figure 6B) suggesting accumulation of oxidized proteins in AD brain.

### Aβ-induced decrease in CREB expression in cultured rat hippocampal neurons

**Figure 7:**
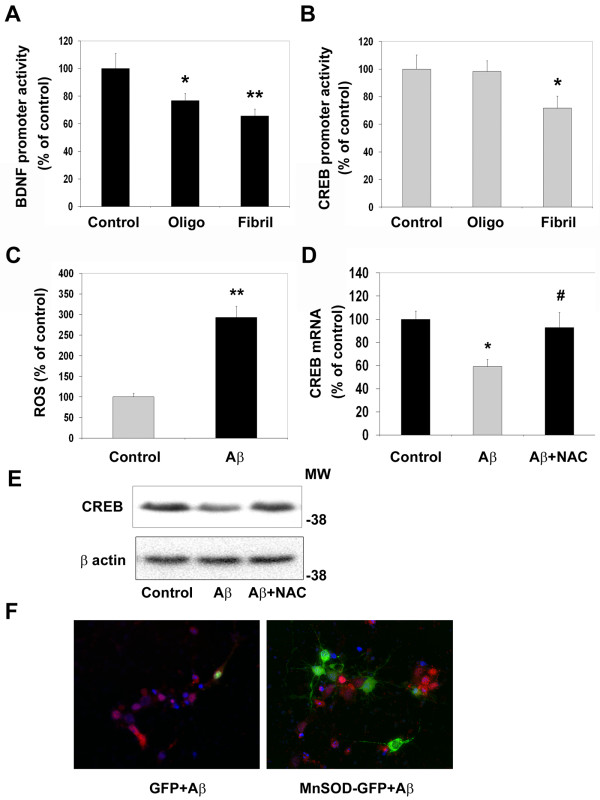
**Effects of Aβ oligomers and fibrils on CREB expression in cultured rat hippocampal neurons**. (A, B) Hippocampal neurons were transfected with luciferase reporters linked to a BDNF promoter (A) or a CREB promoter (B) with LipofectAMINE 2000 reagent. A constitutively active renilla luciferase was also included for transfection efficiency. Transfected cells were treated with Aβ oligomers or fibrils (2 μM) for 24 h. Luciferase activities were determined using dual luciferase assay kit (Promega). Oligomers decreased the activity of BDNF-promoter but not CREB promoter whereas fibrils downregulated both promoters. *p < 0.05 and **p < 0.01 compared to untreated control. (C) Intracellular oxidative stress was measured using an oxidation-sensitive dye, 5- (and 6-)-chloromethyl-2', 7'-dichlorodihydrofluorescein diacetate (CM-H2DCFDA). Rat primary hippocampal neurons were loaded with the dye and the fluorescence emitted at 535 nm was measured using the CytoFluor multiwell plate reader after exposure to Aβ fibrils (2 μM). Aβ fibrils induced significant accumulation of reactive oxygen species in neurons. **p < 0.001 vs control. (D) Neurons were exposed to Aβ fibrils (2 μM) in the presence and absence of N-acetyl cysteine (NAC; 5 mM) for 18 h. Total RNA was isolated and the levels of CREB mRNA were measured by real-time quantitative RT-PCR using a TaqMan™ fluorogenic probe. The values are mean ± SEM of four independent experiments. Aβ-induced downregulation of CREB expression was blocked by the antioxidant, N-acetyl cysteine. *p < 0.01 compared to control and #p0.01 vs Aβ. (E) Neurons were exposed to Aβ fibrils (2 μM) in the presence and absence of N-acetyl cysteine (NAC; 5 mM) for 18 h. Treated neurons were lysed and processed for the Western blot analysis of CREB and β actin. NAC restored Aβ-induced decrease in CREB protein levels. (F) Hippocampal neurons cultured on cover slips were transfected with plasmids encoding GFP or MnSOD-GFP chimeric protein (green) followed by exposure to Aβ (5 μM) for 48 h. The fixed cells were immunostained for the active cleaved fragment of Caspase-9 with Cy-3 (red) as a marker for the mitochondrial pathway of apoptosis. Examination of 20 fields each from 3 independent experiments showed that the cells expressing MnSOD (green) were resistant to Aβ-induced apoptosis.

Although there was correlation between accumulation of oxidative stress markers and decrease of CREB content in Tg2576 mouse brain and AD post-mortem brain, these findings do not suggest a causal relationship between the two. Therefore, we used cultured rat hippocampal neurons to determine the effects of Aβ aggregates on CREB expression. Preincubation of neurons with Aβ fibrils resulted in 40-50% decrease in the activity of CREB-responsive BDNF promoter (p < 0.01) (Figure 7A) and CREB-promoter (p < 0.05) (Figure 7B). Although oligomers decreased BDNF promoter activity (p < 0.05), it had no effect on CREB promoter. Exposure of hippocampal neurons to Aβ fibrils led to generation of reactive oxygen species as shown by the fluorescence of oxidation-sensitive dye (Figure 7C). Aβ treatment also caused 40% decrease (p < 0.01) in CREB mRNA levels and pretreatment with the antioxidant NAC prevented this decrease (Figure 7D). Similar findings were obtained for CREB protein as shown by the Western blot analysis (Figure 7E). The promoter for CREB itself contains CRE sites. Therefore persistent decrease in CREB activity by Aβ fibril-generated oxidative stress could lead to decrease of CREB content itself. To determine if oxidative stress plays a role in Aβ-induced neuronal apoptosis, the antioxidant enzyme MnSOD was overexpressed in rat hippocampal neurons. A chimeric gene consisting of MnSOD and GFP was used in the transfection to identify the neurons expressing this enzyme. The neurons expressing MnSOD were protected from Aβ as shown by significant absence of active caspase-9 staining among GFP-expressing neurons (Figure 7F).

### CREB-mediated protection of rat hippocampal neurons from Aβ-induced apoptosis

**Figure 8:**
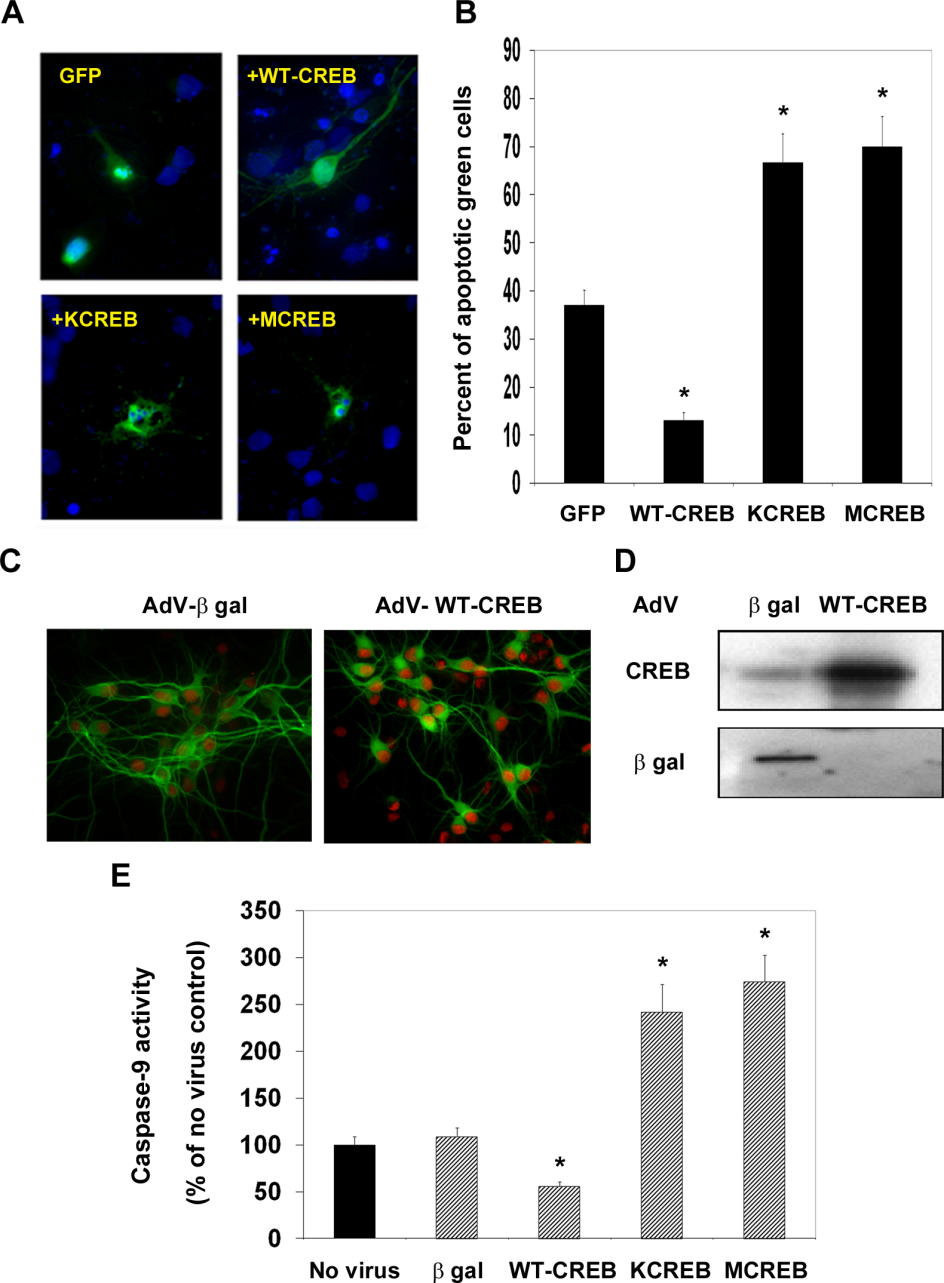
**Neuroprotective effects of CREB**. (A) Rat primary hippocampal neurons cultured on cover slips were transfected with green fluorescent protein (GFP) alone or in combination with wild type CREB or its mutants (KCREB or MCREB). The transfected neurons were exposed to Aβ (5 μM) for 48 h. The apoptotic cells are identified by nuclear condensation with DAPI (Blue). (B) Apoptosis among GFP expressing cells was quantitated by counting neurons with condensed nuclei. At least 500 GFP expressing cells were examined. Neurons were protected by CREB expression and CREB mutants exacerbated Aβ toxicity. *p < 0.01 compared to GFP control. (C) Rat primary hippocampal neurons cultured on cover slips were transduced with recombinant adenoviruses (AdV) encoding β galactosidase (β gal; control) or wild-type CREB (M.O.I: 50) for 48 h. The neurons were fixed, permeabilized, and immunostained for MAP2a (neurites) with FITC (green) and for CREB with Cy3 (red). (D) In addition, cultured rat primary hippocampal neurons were transduced with adenoviral β gal or CREB for 48 h. The whole cell lysates were prepared and immunoblotted for CREB and β galactosidase. Significant upregulation of CREB expression was seen following adenoviral transduction. (E) Cultured rat primary hippocampal neurons were transduced with adenoviruses encoding β galactosidase (β gal), wild type CREB (WT-CREB) or dominant negative mutant forms of CREB (KCREB or MCREB). After 24 h, neurons were exposed to 5 μM of Aβ fibrils for 48 h and then the activity of caspase-9, a marker for the intrinsic pathway of apoptosis was assayed. Significant protection of neurons from Aβ-induced apoptosis was observed. *p < 0.01 vs β galactosidase control.

To test our hypothesis that CREB is a neuroprotective factor, we tested Aβ toxicity in rat hippocampal neurons following modulation of CREB function by plasmid transfection. The wild-type or dominant negative mutant forms of CREB were introduced into cultured neurons along with GFP (to identify the transfected cells; Figure 8A). The transfected neurons were then exposed to Aβ fibrils and apoptotic cells were counted among green cells (Figure 8B). GFP expression did not affect apoptosis induced by Aβ fibrils because nuclear condensation was observed randomly among neurons with and without GFP. Counting of ~500 GFP-expressing neurons showed 64% decrease in apoptotic cells when wild-type CREB was overexpressed. In the case of neurons expressing dominant negative mutant forms of CREB, Aβ-induced neuronal death was exacerbated by 81% (KCREB) and 89% (MCREB). The gene transfer efficiency was moderate (25%-35%) by plasmid transfection in this experiment. Even though we used GFP to identify transfected cells, we sought a more efficient gene transfer method. We used adenoviral gene delivery system to modulate CREB function more efficiently in neurons. First, we tested the efficiency of gene transfer in hippocampal neurons. CREB staining was significantly higher in neurons transduced with adenoviral wild-type CREB (Figure 8C). Western blot analysis showed a 3-fold increase in CREB levels (Figure 8D). Next, the neurons transduced with adenoviral β gal (control) CREB, KCREB and MCREB were exposed to Aβ fibrils and the activity of caspase-9, a marker for the intrinsic mitochondrial pathway of apoptosis was determined (Figure 8E). Adenoviral β gal did not significantly increase the basal caspase-9 activity seen in neurons that were not transduced with a viral vector. Transduction of neurons with wild type CREB resulted in 42% decrease in caspase-9 activity whereas downregulation with two mutant forms of CREB resulted in exaggeration of caspase-9 activation by 140-170%. These observations suggest that the preservation of CREB function is essential to protect hippocampal neurons from Aβ-induced injury.

## Discussion

CREB is a constitutively expressed nuclear transcription factor needed for cognition and neuronal survival. Its regulation is primarily through phosphorylation at serine 133 by several signaling kinases and thus responds to a variety of extracellular signals [10,13,25,26]. CREB-regulated gene expression has been shown to be downregulated in AD brain. In this study, we show that the expression of CREB itself is decreased in Tg2576 mouse hippocampal neurons, AD post-mortem hippocampal samples and in Aβ-treated rat hippocampal neurons. Our findings suggest that persistent downregulation of CREB-regulated gene expression could lead to decrease in the levels of CREB itself which could exacerbate neurodegeneration in AD.

Tg2576 mice overexpressing human APP with the Swedish mutation are characterized by Aβ deposition and cognitive dysfunction [27,28]. Previous studies have suggested that markers of CREB function are downregulated in the hippocampal neurons of this mouse model [14,15,29]. We demonstrate in the present study by laser capture microdissection (LCM) that the levels of CREB mRNA are significantly decreased in the hippocampal neurons of Tg2576 mouse brain (Figure 1). The expression of BDNF, a target gene of CREB was also found to be reduced in LCM-captured neurons. Further examination of CREB protein by immunohistochemical analysis showed age-dependent decreases in hippocampal neurons, more significantly in the transgenic mouse brain (Figure 2). Markers of oxidative stress including HNE and MDA were also elevated. We observed decreases in CREB levels especially in the regions where astrocytes were abundantly present (Figure 3E). Astrocytosis is known to play a role in neurodegeneration through release of cytokines [30,31]. We have previously reported in cultured MIN6 cells, a mouse pancreatic cell line that a combination of proinflammatory cytokines, IL-1β, TNF-α and IFN-γ decreases CREB expression following chronic exposure [32].

Oxidative stress is known to play an important role in the neurodegenerative process of Alzheimer's brain. Accumulation of free radicals is an important feature of aging, which is also a risk factor for AD. Markers of oxidative stress are found in aged rats, especially in those with impaired spatial learning [33]. Hensley et al. demonstrated that Aβ aggregation leads to generation of oxidative stress *in vitro *[34,35]. In addition, several other agents, including iron, aluminum, and advanced glycosylation end products, induce oxidative stress in AD [36-38]. Lipid peroxidation and DNA oxidation products accumulate in AD brains as a result of oxidative stress [36,39-41]. We observed increased protein-bound HNE and protein oxidation in AD post-mortem samples as shown by Oxyblot analysis (Figure 6). SDS-extractable Aβ accumulation showed inverse correlation with CREB levels in AD post-mortem brain (Figure 4E). These findings suggest that Aβ-generated oxidative stress could play a role in downregulation of CREB expression. To determine the direct effects of Aβ on CREB expression we examined the activity of a luciferase reporter gene driven by the CREB promoter in cultured rat hippocampal neurons. CREB promoter activity was downregulated by Aβ fibrils but not by Aβ oligomers (Figure 7B). The oligomers did decrease the activity of CREB-dependent BDNF promoter activity (Figure 7A). Furthermore, Aβ fibril-induced decrease in CREB mRNA levels was prevented by preincubation of neurons with the antioxidant, NAC (Figure 7D). Thus our findings with cultured hippocampal neurons provide direct evidence for the downregulation of CREB expression by oxidative stress. Although Aβ oligomers did not play a role in the downregulation of CREB expression in this study, their role in AD pathology is well characterized.

Several reports have shown that caspase activation, a marker of apoptosis, is observed in the neurons in AD brain (reviewed in [42]). Caspase activation can also play a role in AD pathology independent of causing neuronal death. For example, caspase-cleaved tau undergoes conformational changes that facilitate filament formation, and Aβ localizes to cleaved tau [43]. Although significant loss of neurons is observed in human AD brain, marked neuronal apoptosis is not observed in mouse models of AD. We observed modulation of several proteins in the apoptosis pathway in AD post-mortem brain (Figure 5). The levels of anti-apoptotic Bcl-2 and caspase inhibitors, BIRC3 and BIRC4 decreased and proapoptotic Bax increased in AD brain leading to activation of caspase-9, a marker for the intrinsic pathway of apoptosis. CREB plays an important role in increasing the levels of proteins that maintain mitochondrial membrane integrity and prevent the release of cytochrome c, an activator of caspase-9. Oxidative stress is known to induce neuronal apoptosis by a dual mechanism of downregulation of anti-apoptotic genes and upregulation of proapoptotic genes [44].

We have previously reported that IGF-I induces CREB-mediated bcl-2 expression in PC12 cells through multiple signaling pathways [12,13]. We also reported that oxidative stress downregulates bcl-2 expression in rat hippocampal neurons [23]. In the present study, we observed that Aβ-induced neuronal apoptosis is exaggerated when dominant negative mutant forms of CREB (KCREB and MCREB) were overexpressed along with GFP by plasmid transfection (Figure 8A). KCREB is mutated at the DNA binding region of CREB. This mutant will heterodimerize with endogenous CREB and sequester it away from target promoters. MCREB is mutated at the critical phosphorylation site (S133A). MCREB will bind to CRE but cannot bind to the coactivators. We also tested the effects of modulation of CREB function by adenoviral transduction on Aβ toxicity (Figure 8E). Apoptosis by the intrinsic pathway was induced by Aβ as shown by the activation of caspase-9 as observed in AD post-mortem brain (Figure 5). Expression of wild type CREB decreased neuronal death induced by Aβ significantly. There was also exacerbation of Aβ-induced apoptosis when the neurons were transduced with adenoviral KCREB or MCREB. Thus chronic CREB downregulation caused by oxidative stress could be an important cause of loss of neurons in AD brain.

## Conclusions

The primary events occurring in the pathogenesis of AD include the deposition of Aβ-containing senile plaques and the formation of neurofibrillary tangles. This triggers a slow neurodegenerative process involving multiple insults to neurons, including free radical generation and inflammatory responses. During this slow progressive phase, the cellular defense mechanism fails, due to oxidative stress-induced changes in transcriptional events including downregulation of CREB expression. In the treatment of AD, downstream interventions cannot be discounted as it is essential to repair neuronal injury while attempting to help clear Aβ accumulation. Small molecule enhancers of CREB-mediated gene expression are being considered as potential therapeutic agents in the treatment of AD [45].

## Methods

### Materials

Cell culture media and supplies were purchased from Gemini BioProducts (Woodland, CA, USA) and Invitrogen-Life Technologies (Carlsbad, CA). Antibodies to CREB, phosphorylated form of CREB, synaptophysin, BIRC3, BIRC4, Bax, active caspase-9, GFAP, and β actin were from Cell signaling (Beverly, MA). The antibodies to HNE and MDA and caspase-9 assay kit were obtained from Millipore (Billerica MA). The cDNA encoding a chimeric protein for GFP and MnSOD was provided by Dr. Sonia Flores (University of Colorado, Aurora, CO). IgG linked to Cy3 or FITC were obtained from Jackson ImmunoResearch (West Grove, PA, USA). All other fine chemicals were from Sigma (St. Louis, MO). We obtained 24 post-mortem samples (12 each of control and AD) from the AD Brain bank at University of Colorado. The mean age was 75 y for AD and 73 y for controls. The postmortem delay (PMD) was 3-12 h (mean 7 h). This is comparable to PMD reported in other studies [46-49]. The groups of subjects were characterized as AD or normal controls using the criteria from the Consortium to Establish a Registry for AD [50].

### Laser capture microdissection of mouse hippocampal neurons

All animal procedures were performed with the approval of the Subcommittees on Research Safety and Institutional Animal Care and Use at the Denver Department of Veterans Affairs Medical Center, Eastern Colorado Health Care System (ACORP# 08006M). Tg2576 mice and nontransgenic controls were anesthetized and perfused with cold RNase-free phosphate buffered saline. The brain was removed and embedded in Tissue-TeK OCT in a plastic mold and frozen. Frozen brain sections (10 μM) were cut on a cryostat and collected on Superfrost slides (Fischer Scientific, Pittsburgh, PA). DEPC-treated water was used in all procedures. All solutions except wash buffers contained 0.5 U/μl RNase inhibitor. Brain sections (10 μM) were stained using HistoGene™ Stain, (Arcturus) to identify the neurons. LCM was performed using AutoPix LCM system from Arcturus Engineering (Mountain View, CA). The neurons in CA1, CA3 and dentate gyrus regions were selectively captured from each section onto Capsure LCM macro caps. Total RNA was isolated from LCM samples using a PicoPure RNA isolation kit (Arcturus). Because the RNA yield is low, additional amplification steps were used following the RiboAMP RNA amplification kit instructions from Arcturus. Real time RT-PCR analysis using Taqman probes was performed for mouse neurofilament heavy chain (mNFHc) to determine the neuronal nature of the samples and human APP to differentiate the RNA samples isolated from nontransgenic and APP transgenic mice. In addition, the mRNA levels of CREB and BDNF were compared in amplified RNA samples from nontransgenic and transgenic mice. The PCR reactions in 96 wells were monitored in real time in an ABI Prism 7700 sequence detector (Perkin Elmer Corp./Applied Biosystems).

### Immunohistochemical analysis of Tg2576 mouse brain

The tissue sections were de-paraffinized and hydrated through serial dilutions of ethanol and then in distilled water. The hydrated sections were steamed in 10 mM sodium citrate buffer (pH 6.0) for 30 min for antigen retrieval. They were treated with 1% hydrogen peroxide for 10 min and blocked with 5% horse serum for 1 h at RT. Subsequently, depending on whether the antibody was from mouse or rabbit, we used a 'mouse on mouse' or rabbit ABC elite kit from Vector laboratories (Burlingame, CA), respectively. The chromogens were Novared and/or DAB. Counterstaining was with methyl green. For double immunostaining, color was developed with the first primary antibody followed by incubation with the second antibody and color development.

### Western blot analysis

Proteins were resolved by SDS-PAGE electrophoresis, transferred to polyvinylidene difluoride membranes and blocked with 5% non-fat dry milk for 1 h at RT. The membranes were exposed to primary antibodies at a dilution of 1:1000 overnight at 4°C. After washing with 5% non-fat milk, the membranes were incubated in the presence of appropriate secondary antibodies linked to alkaline phosphatase for 1 h at RT. After treating with alkaline washing buffer (10 mM Tris-HCl (pH 9.5), 10 mM NaCl and 1 mM MgCl_2_), signals were developed with CDP-Star reagent (New England Biolabs, Beverly, MA) and exposed to X-ray film. The intensity of bands were measured by using Fluor-S MultiImager and Quantity One software (Bio-Rad, Hercules, CA) and corrected for the levels of β actin. **Oxyblot analysis**: Oxidative modification of proteins by free radicals was determined for AD-post mortem samples along with age-matched controls using a kit from Millipore (Billerica, MA). The protein samples (20 μg) in 5 μl were mixed with 5 μl of 12% SDS for a final concentration of 6% and derivatized with 10 μl of 1X DNPH solution. The samples were incubated at RT for 15 min, neutralized and electrophoresed. Following transfer, the blots were immunoprobed with anti-DNP antibody and the signals were developed with CDP-Star.

### Aβ assay and preparation of Aβ aggregates

After extracting the soluble fractions from post-mortem samples with Tris-buffered saline (pH 7.6) containing 1% Triton X-100 and protease inhibitors, the tissue pellets were extracted with 2% SDS containing protease inhibitors. The extracts were appropriately diluted and Aβ 1-42 levels were determined by sandwich ELISA. For capture, the Ban-50 (1-42) antibody was used and the BC-05 antibody was used for detection. To determine Aβ-induced toxicity in cultured neurons, Aβ aggregates were prepared by the following procedure: Aβ (1-42) peptide purchased from Peptide 2.0 (Chantilly, VA) was first monomerized by resuspending in hexafluoroisopropanol followed by incubation at RT for 1 h. After evaporation of solvent under vacuum, Aβ is dissolved in DMSO and stored at -20°C. Aβ oligomer stock (100 μM) was prepared by incubation in F-12 (without phenol red) culture medium at 4°C for 24 h. Aβ fibril stock (100 μM) was prepared by incubation in HCl (10 mM) at 37°C for 24 h.

### Culture of rat hippocampal neurons and transfection

Hippocampal neurons were isolated from E18 rat embryos obtained from timed-pregnant Sprague-Dawley rats as previously described [51]. The astrocyte population was less than 5%. The neurons were cultured in serum-free Eagle's medium containing 2% antioxidant-free B27 supplement (Life Technologies). The experiments were carried out in these neurons after one week in culture [22]. Total RNA was isolated from treated neurons using an isolation kit (Versagene RNA; Fisher Scientific, Pittsburgh, PA). Transient transfection of rat hippocampal neurons cultured in 12 well dishes to about 70% confluence was performed using LipofectAMINE 2000 reagent (Invitrogen-Life Technologies) according to the procedure described previously [22]. BDNF promoter and CREB promoter linked to a luciferase reporter gene were provided by Anne West (Harvard Medical School, Boston) and Dr. Dwight Klemm (University of Colorado) respectively. A constitutively active renilla luciferase (pRL-TK-luc) was included to correct for transfection efficiency and nonspecific actions of oxidative stress. After 6 h of transfection, the neurons were exposed to Aβ oligomers or fibrils for 18 h. The cells were processed for the assay of luciferases using dual luciferase assay kit (Promega). The transfection efficiency for rat hippocampal neurons using LipofectAMINE 2000 reagent was ~30%.

### Immunocytochemistry

Rat hippocampal neurons were cultured on glass cover slips (Carolina Biological Supply, Burlington, NC) prepared according to the methods of Goslin and Banker [52]. Treated neurons were fixed in 4% paraformaldehyde for 30 min at RT and washed with PBS. The cells were permeabilized in PBS containing 0.2% Triton X-100 and 5% BSA for 90 min at RT, followed by incubation with the primary antibody at 4°C overnight. Double immunostaining was performed using monoclonal and polyclonal antibodies for two targets. After washing with PBS, appropriate secondary antibodies linked to Cy3 and FITC were added along with DAPI (2 μg/ml; nuclear staining) for 90 min at RT. The cells were then washed in PBS, mounted on slides and examined by digital deconvolution microscopy.

### Measurement of intracellular oxidative stress

Intracellular ROS (reactive oxygen species) generation was measured in neurons exposed to Aβ aggregates using an oxidation-sensitive dye, 5- (and 6-)chloromethyl-2',-7'-dichlorodihydrofluorescein diacetate (CM-H2DCFDA). When the cells are loaded with this dye, its acetate groups are cleaved by cellular nonspecific esterases, trapping the dye in the cell. Although this "leuco" form of the dye is colorless, the oxidized dye fluoresces at 535 nm when excited with 485 nm light. The cells were loaded with the dye and the fluorescence was measured using the CytoFluor multiwell plate reader.

#### Caspase-9 assay

Cultured rat hippocampal neurons transduced with adenoviruses followed by exposure to Aβ fibrils were processed for the assay of caspase-9 using a kit from Millipore (Billerica, MA). Supernatant of the cell lysates were incubated in the presence of substrate, *p*-nitroanilide-labeled LEHD. The released chromophore was read at 405 nm using a microplate reader.

### Statistical analysis

Data presented are Mean ± SE. Statistical evaluation was performed by one-way ANOVA with Dunnett's multiple comparison test.

## Abbreviations

**AD: **Alzheimer's disease; **AMP: **Adenosine monophosphate; **BDNF: **Brain-derived neurotrophic factor; **CBP: **CREB binding protein; **CREB: **Cyclic AMP response element binding protein; **GFP: **Green fluorescent protein; **HNE: **4-hydroxynonenal; **IGF-I: **Insulin-like growth factor-I; **LCM: **Laser capture microdissection; **MDA: **Malone dialdehyde; **NAC: **N-acetyl cysteine; **RT-PCR: **Reverse transcription-polymerase chain reaction

## Competing interests

The authors declare that they have no competing interests.

## Authors' contributions

SP designed the experiments, performed the laser capture microdissection and wrote the manuscript. MW performed immunohistochemical analysis and experiments with cultured rat hippocampal neurons. TM performed Western blot analysis and contributed in other experimental procedures. CIS provided the AD post-mortem brain samples. CBE was a consultant for experiments with Tg2576 mouse brain. All authors have read and approved the final manuscript.
